# Understanding home psychiatric nursing: a phenomenological study on nurses’ challenges, emotional resilience, and professional growth in Jeddah, Saudi Arabia

**DOI:** 10.3389/fpubh.2026.1775451

**Published:** 2026-03-09

**Authors:** Amal I. Khalil, Sawsan K. Elgalad, Abeer S. Esawi

**Affiliations:** 1 King Abdullah International Medical Research Center, Jeddah, Saudi Arabia; 2 College of Nursing, King Saud bin Abdulaziz University for Health Sciences, Jeddah, Saudi Arabia; 3 Ministry of National Guard Health Affairs (MNGHA), Jeddah, Saudi Arabia; 4 Faculty of Nursing, Menoufia University, Shibin El-Kom, Egypt; 5 Faculty of Nursing, Alexandria University, Alexandria, Egypt; 6 Faculty of Nursing, Cairo University, Cairo, Egypt

**Keywords:** emotional resilience, home psychiatric nursing, phenomenology, professional growth, qualitative study, Saudi Arabia

## Abstract

**Background:**

Home-based psychiatric nursing involves distinct challenges compared to inpatient care, including unpredictable care environments, complex family dynamics, and heightened emotional demands. Understanding nurses’ lived experiences is essential for strengthening workforce readiness, resilience, and professional development, particularly in Saudi Arabia where community mental health services are expanding.

**Aim:**

This study explored the lived experiences of home psychiatric nurses in Jeddah, Saudi Arabia, focusing on perceived challenges, coping strategies, emotional resilience, and professional identity development.

**Methods:**

A qualitative interpretive phenomenological design, guided by Van Manen’s phenomenology, was employed to examine nurses’ lived experiences. Resilience Theory informed the exploration of coping mechanisms and emotional adaptation, while Meleis’ Transition Theory provided a conceptual lens for understanding nurses’ professional role transitions from hospital-based to home-based psychiatric care. Semi-structured interviews were conducted with 20 home psychiatric nurses, and data were thematically analyzed to identify patterns of meaning across participants’ narratives.

**Results:**

Five interrelated themes emerged: (1) Entering Home Psychiatric Care: Preparedness, Training Gaps, and Developing Autonomy; (2) Navigating the Complexity of Home Environments and Workload Demands; (3) Emotional Labor, Compassion Fatigue, and Coping Strategies; (4) Ethical Dilemmas, Safety Risks, and Limited Institutional Support; and (5) Professional Growth, Identity Transformation, and Meaningful Engagement.

**Conclusion:**

Home psychiatric nursing requires competencies beyond traditional hospital-based training, particularly in resilience, ethical decision-making, and adaptive professional practice. Organizational support, targeted training, and clear safety frameworks are critical for sustaining nurses’ well-being and optimizing patient care. These findings offer theoretically informed insights to guide policy, education, and service development in community mental health care in Saudi Arabia.

## Background

In recent years, there has been a marked increase in the demand for psychiatric care delivered in home and community settings. This shift has been driven by global mental health reforms emphasizing deinstitutionalization, reduced reliance on inpatient care, efforts to minimize stigma associated with hospital-based mental health services, and the growing need for continuous, person-centered care within patients’ everyday environments (1, 2). International evidence indicates that home-based psychiatric services can enhance continuity of care, strengthen therapeutic relationships, and support recovery by addressing psychosocial needs within patients’ natural contexts—benefits that are less readily achievable in inpatient or outpatient clinic settings (3–5).

Despite these advantages, psychiatric nursing in home settings presents a distinct practice context that differs fundamentally from inpatient psychiatric units and outpatient clinics. Unlike institutional environments—where care is delivered within structured systems supported by multidisciplinary teams, standardized protocols, and immediate access to clinical and security resources—home psychiatric nurses often work independently, without real-time professional backup. International studies from Europe, North America, and Asia consistently report that nurses in home-based psychiatric care face professional isolation, unpredictable patient behavior, safety risks, ethical dilemmas, and heightened emotional demands, all within uncontrolled and variable home environments (6–9). These challenges are qualitatively different from those encountered in inpatient settings, where risks are mitigated through environmental control, team-based decision-making, and rapid escalation pathways.

Research from Australia, Sweden, and the United Kingdom further highlights that home psychiatric nursing requires advanced competencies in autonomous clinical judgment, risk assessment, crisis management, and family negotiation, often exceeding the scope of traditional psychiatric nursing education (10–12). In the home setting, nurses must simultaneously manage clinical care, emotional support, safety assessment, and family dynamics during a single visit, frequently with limited resources. When role boundaries are unclear or organizational support is insufficient, these demands can contribute to emotional fatigue, compassion fatigue, and role strain (13–15).

At the same time, international literature suggests that home psychiatric nursing can also serve as a context for professional growth, fostering autonomy, holistic practice, and emotional resilience—particularly when nurses are supported through reflective practice, peer networks, and clear organizational frameworks (16–18). Importantly, resilience in this context is increasingly understood not as a fixed individual trait, but as a dynamic process shaped by work environment, institutional resources, and professional role clarity (19).

In Saudi Arabia, home psychiatric nursing remains an emerging field, and empirical research examining nurses’ lived experiences within this specific context is limited. Although the Ministry of Health has increasingly prioritized mental health services and home care models as part of Vision 2030—aimed at improving access, reducing stigma, and strengthening community-based care—existing research has largely focused on inpatient psychiatric services or general home healthcare, rather than psychiatric home nursing specifically (20, 21). This gap is particularly evident in major urban centers such as Jeddah, where rapid population growth and increasing mental health needs place additional demands on community-based services.

As the global burden of mental illness continues to rise—with depression, anxiety disorders, bipolar disorder, and psychotic disorders among the leading causes of disability worldwide (22)—psychiatric nurses are playing an increasingly central role in sustaining community and home-based care. However, without a nuanced understanding of nurses’ experiences in these roles, healthcare systems risk inadequate workforce support, leading to burnout, high turnover, and compromised quality of care (23, 24).

The core research phenomenon guiding this study is the lived experience of professional meaning-making and emotional resilience among psychiatric nurses delivering care independently in patients’ homes. Specifically, this study seeks to understand how nurses interpret and adapt to the emotional, ethical, and professional challenges inherent in home-based psychiatric care, and how these experiences shape their professional identity and commitment over time.

To explore this phenomenon, the study adopts a phenomenological research design. Phenomenology is a qualitative approach that focuses on how individuals perceive, interpret, and assign meaning to their lived experiences, prioritizing depth and subjective understanding rather than generalization (25). Given the complex emotional, professional, and ethical dimensions of home psychiatric nursing, this approach is particularly well suited to capturing the nuanced realities of nurses’ work in this distinctive care environment.

By engaging closely with participants’ narratives, this study aims to illuminate how psychiatric nurses in Jeddah navigate uncertainty, develop resilience, and construct professional identity within home-based care. These insights contribute to the limited Saudi literature while also extending international understanding of community mental health nursing, offering culturally grounded evidence to inform workforce development, organizational support, and policy initiatives.

### Significance of the study

Home psychiatric nursing is vital for providing mental health services in community environments, maintaining care continuity, and decreasing hospital admissions (26). Nonetheless, nurses in this area encounter distinct difficulties, such as emotional pressure, safety issues, and professional solitude (27). This research is important in tackling these concerns by investigating nurses’ real-life experiences, emphasizing their obstacles, emotional strength, and career development in Jeddah, Saudi Arabia. Recognizing these factors can lead to policies that promote nurse well-being, alleviate burnout, and boost job satisfaction (28). Moreover, findings from this research can guide training initiatives and workforce development plans, guaranteeing that home psychiatric nurses obtain sufficient support to provide efficient patient care (29). This study can boost psychiatric patient outcomes by enhancing working conditions and resilience strategies, leading to stronger relationships between nurses and patients while encouraging adherence to treatment (30). In addition, it will contribute to the expanding research in psychiatric nursing, offering evidence-based guidance for enhancing home-centered mental health services in Saudi Arabia and elsewhere.

### Theoretical framework

**Figure 1:**
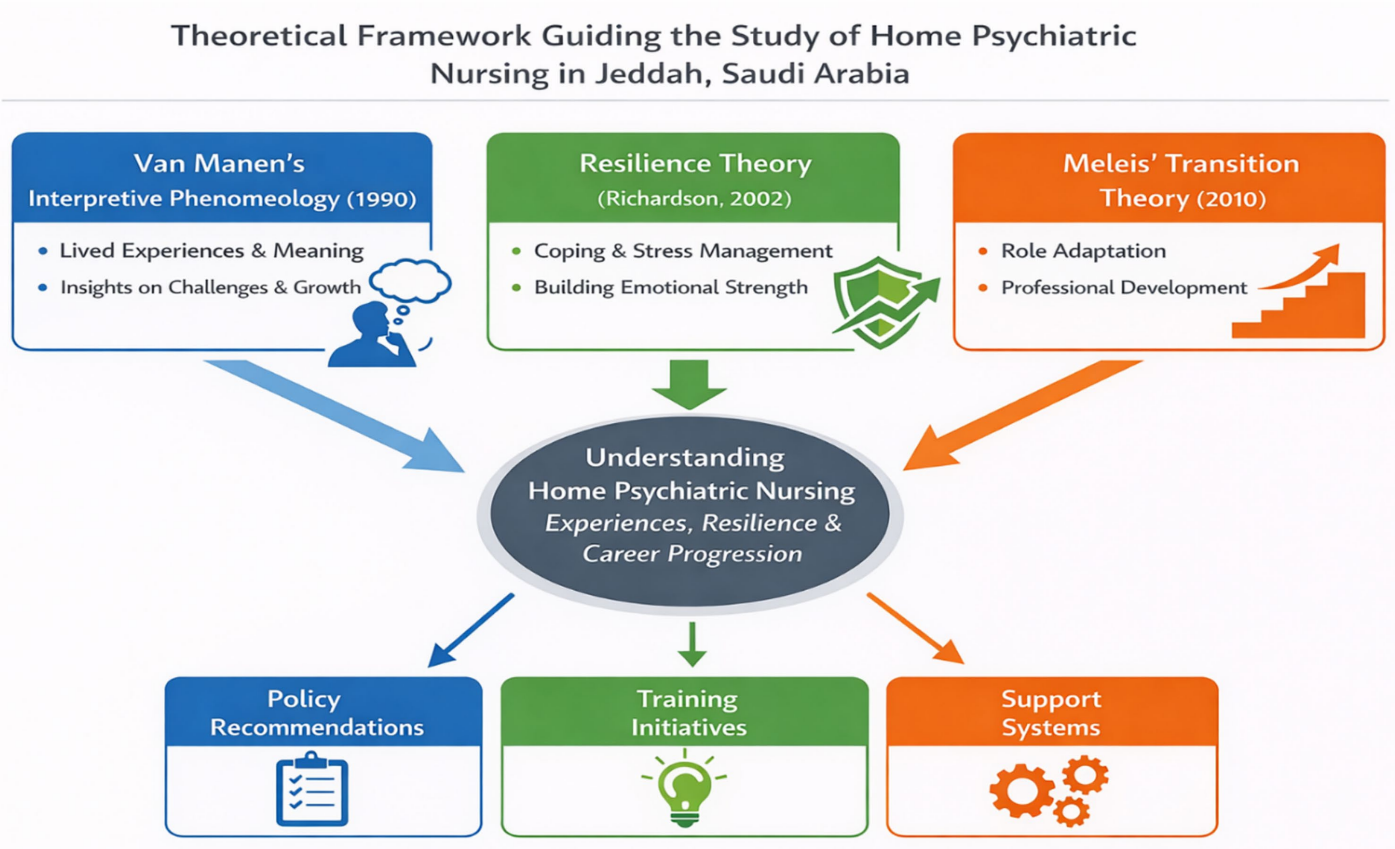
Theoretical framework guiding the study of lived home nurses’ experiences.

This phenomenological study is informed by Van Manne’s Interpretive Phenomenology, Resilience Theory, and Meleis’ Transition Theory, which together provide a comprehensive lens to explore the lived experiences of psychiatric nurses working in home settings in Jeddah, Saudi Arabia. Van Manne’s Interpretive Phenomenology (31) plays a crucial role in this research by enabling an in-depth exploration of the meaning behind nurses’ experiences, emphasizing their personal insights into challenges, emotional resilience, and career growth. This approach ensures that the study captures the profound, personal experiences of nurses engaged in home psychiatric care. Additionally, Resilience Theory (32) offers a framework for understanding how nurses handle emotional stress and challenges in their roles. It highlights the dynamic nature of resilience, identifying protective factors that assist nurses in managing stress, preventing burnout, and maintaining professional commitment. Lastly, Meleis’ Transition Theory (33) provides insights into how nurses navigate role transitions, adapt to new challenges, and undergo professional development while working in the complex and often unpredictable environment of home psychiatric care. By integrating these frameworks, this research ensures a thorough understanding of how nurses face challenges, build resilience, and advance in their careers, offering valuable insights that can inform policies, training programs, and support systems to enhance psychiatric home care services (Figure 1).

**General Aim:**

To explore and investigate the real-life experiences of home psychiatric nurses in Jeddah, Saudi Arabia, with a focus on the obstacles they face, their emotional resilience, and their professional development within the realm of home-based mental healthcare.

**Specific Objectives:**

- To identify the difficulties encountered by home psychiatric nurses in providing mental health care.
- To delve into the coping strategies and resilience mechanisms that nurses use to handle emotional and professional stress.
- To assess the influence of home psychiatric nursing on the professional growth and career advancement of nurses.
- To explore how home psychiatric nurses manage role transitions and adapt to community-based psychiatric care.
- To offer recommendations for policies, training, and support systems that improve the well-being and effectiveness of home psychiatric nurses.

## Methodology

### Research design

This study employed a qualitative interpretive phenomenological design, guided by Van Manen’s hermeneutic phenomenology, to explore the lived experiences of psychiatric nurses providing care in home-based settings. Interpretive phenomenology is well suited to this inquiry, as it seeks to understand how individuals make meaning of their experiences within specific social, cultural, and professional contexts, rather than merely describing observable phenomena (25, 53).

The central focus of the study was the phenomenon of professional meaning-making and emotional resilience among home psychiatric nurses, examined through their subjective accounts of challenges, coping processes, and professional identity development. Consistent with phenomenological inquiry, the analysis prioritized shared meanings while remaining attentive to variations in individual experiences that enriched thematic interpretation.

### Setting and participants

The study was conducted at the Erada Complex for Mental Illness Services, affiliated with the Ministry of Health in Jeddah, Saudi Arabia, and focused on nurses who provided home psychiatric care services across various home-based mental health settings for individuals with psychiatric disorders. The study population included all home psychiatric nurses working at the Erada Complex, from whom a purposive sample of 20 out of 28 participants was selected to ensure diversity in experience levels, gender educational background, professional experience, and marital status to provide context for interpreting participants’ experiences and potential influences on resilience and professional identity. “.

Eligible participants were licensed psychiatric nurses who were actively engaged in home-based mental health care, had at least 1 year of experience in home psychiatric nursing, and were willing to participate and share their lived experiences. Nurses with less than 1 year of experience in home psychiatric care, those not directly involved in patient care (such as administrative staff), and those who did not provide consent were excluded from the study.

### Sample size justification

In total, 20 psychiatric nurses took part in the study. The sample size was determined by the concept of information power, which posits that fewer participants are needed when the data is more relevant and comprehensive (34). Recruitment continued until experiential saturation was achieved, meaning no new themes or significant insights were identified in further interviews. The final number of participants aligns with methodological guidelines for phenomenological research and is similar to other qualitative studies in the fields of psychiatric and community nursing.

### Home psychiatric nursing service model

In Jeddah, psychiatric nursing services at home are mainly provided through planned visits as part of community mental health care. Participants noted that the timing and length of these visits are not predetermined but are instead adjusted based on the patient’s clinical status, behavioral stability, and the cooperation level of both the patient and their family. Nurses indicated that visits could last anywhere from about 15 min to a maximum of 45 min. Shorter visits are typically conducted when patients are agitated or aggressive, while longer visits occur when patients are calm, cooperative, and need more extensive care. The services often include administering medications (like long-acting injectable antipsychotics), monitoring mental status, providing brief psychoeducation, and coordinating with family members. This adaptable service model greatly influences nurses’ professional experiences, emotional demands, and safety considerations in home-based psychiatric care.

## Data collection and analysis

### Interview schedule development

The interview guide was developed based on Van Manen’s Interpretive Phenomenology, Resilience Theory, and Meleis’ Transition Theory to ensure that each dimension and question captures the core constructs relevant to our study. Specifically, the nine dimensions were chosen to explore home psychiatric nurses’ lived experiences, including professional challenges, emotional resilience, coping strategies, transitions in care roles, and professional growth. The 22 questions were carefully designed to provide in-depth insight into these areas while remaining manageable for participants. A semi-structured interview schedule was crafted following a thorough examination of existing literature on home psychiatric nursing, community mental health care, emotional resilience, and professional identity (5, 10, 16). This schedule included nine key dimensions aimed at investigating the study’s goals:

- Demographic and professional background
- Reasons for choosing home psychiatric nursing
- Professional readiness and training
- Role expectations and professional identity
- Interactions with patients and families
- Emotional and psychological challenges
- Coping mechanisms and resilience
- Views on organizational support
- Professional development and career path

The schedule underwent a pilot test with three psychiatric nurses who were not part of the final sample to evaluate its clarity, relevance, and practicality. Based on feedback from the pilot, minor adjustments were made to the wording and order of questions to improve understanding and ensure that the questions prompted open-ended, reflective answers.

### Data collection comprehensive procedure

With the consent of the participants, all interviews were recorded and transcribed word-for-word for analysis. To maintain authenticity and cultural context, the recordings were first transcribed in Arabic and then translated into English. A bilingual expert verified the translation’s accuracy and cultural nuances to ensure linguistic and contextual fidelity (35). Participants were given the opportunity to review the translated transcripts to confirm the accuracy and meaning of their responses, thereby enhancing the data’s credibility (35, 36). Trustworthiness and credibility were further bolstered through systematic peer debriefing, where the research team and external qualitative researchers engaged in reflective discussions to minimize bias and promote analytical rigor (36). Data triangulation was achieved by integrating interview transcripts, field notes, and direct observational notes to ensure consistency and depth of interpretation (35). Transferability was supported by providing rich, detailed descriptions of the research context, setting, and participant characteristics, facilitating applicability to similar healthcare environments. Reflexivity was maintained using reflective journals, allowing the research team to document and critically examine personal assumptions and potential biases throughout the analytic process (36).

### Determination of data saturation

Data saturation was assessed through a process of iterative and simultaneous data collection and preliminary analysis, aligning with phenomenological qualitative research methods. After each interview, the transcripts were examined and subjected to initial thematic analysis following Van Manen’s hermeneutic approach.

Around the 16th interview, the research team noticed a recurrence in the descriptions of experiences, with no new meanings, codes, or thematic patterns emerging. To verify this, four more interviews were conducted. These additional interviews did not reveal any new themes or conceptual insights, confirming that saturation had been reached.

Saturation was thus defined as the point where: No new experiential themes appeared, Existing themes were thoroughly detailed and internally consistent, Additional data added depth but not novelty. This approach guaranteed adequate depth, reliability, and thoroughness of the results, justifying the final sample size of 20 participants.

### Data analysis

Interviews were audio-recorded, transcribed verbatim, and analyzed using a combination of Colaizzi’s phenomenological method (25) and Interpretative Phenomenological Analysis (IPA) guided by Van Manen’s hermeneutic phenomenology. Although the interview schedule included nine guiding dimensions, the analysis was inductive: themes and subthemes emerged directly from participants’ narratives without imposing predefined categories. The guiding dimensions served only as prompts to structure interviews and did not constrain the analytic process.

**Figure 2:**
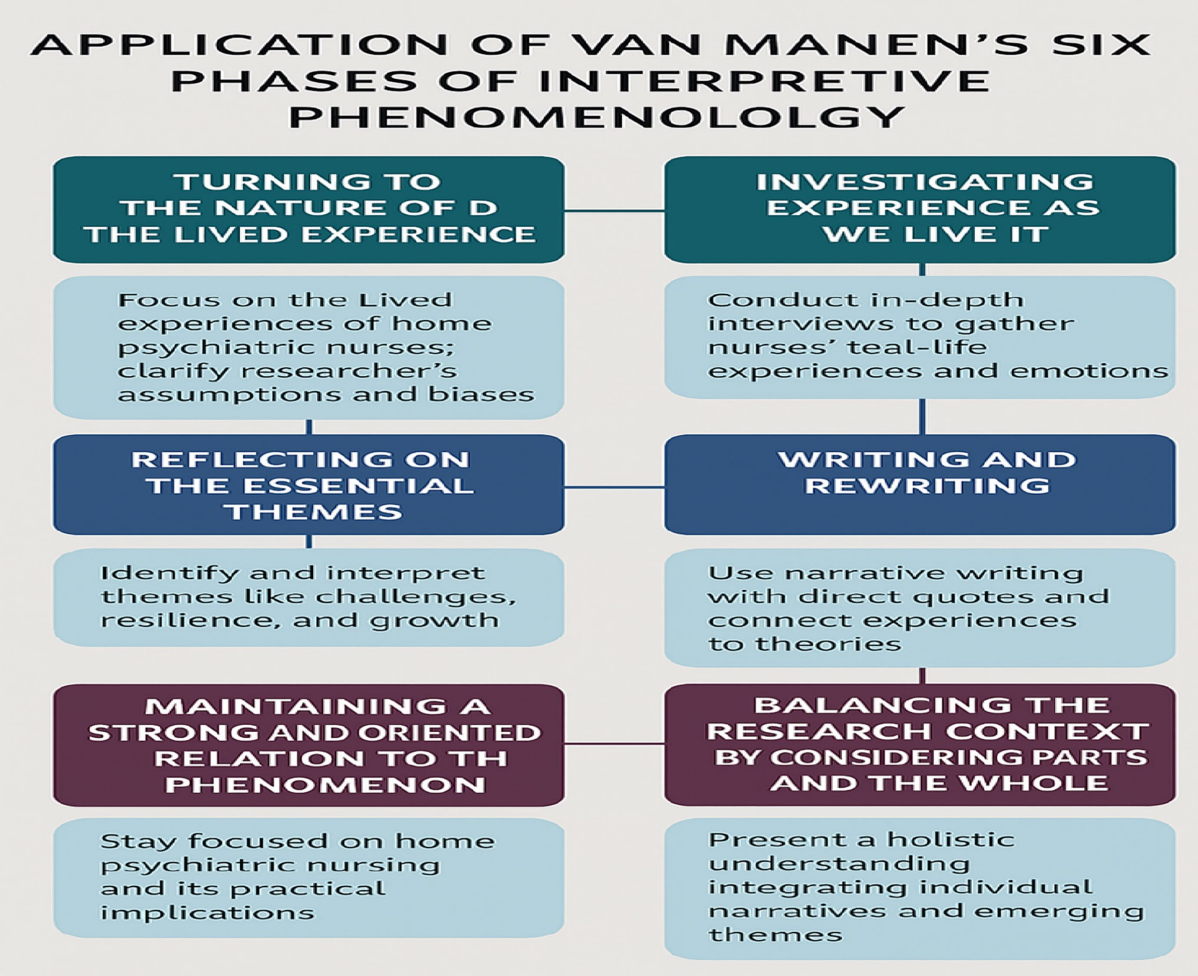
Six Van Mann’s phases of data analysis.

Van Manen’s six-phase approach was systematically applied to gain a deep understanding of nurses’ lived experiences, including how they navigated occupational challenges, developed resilience, and experienced professional growth within home-based psychiatric care (Figure 2). This theoretically informed framework ensured that the analysis captured both explicit and implicit meanings in participants’ narratives, producing rich, nuanced, and contextually grounded insights relevant to public health practice and nursing research.

To maintain methodological rigor in this phenomenological research, we implemented several strategies. To bolster trustworthiness, our analysis included triangulation, peer debriefing, and member checking. Triangulation involved gathering data through comprehensive semi-structured interviews with 20 home psychiatric nurses from varied backgrounds and experiences to capture a range of perspectives.

For peer debriefing, we engaged two seasoned qualitative researchers to discuss initial coding and theme development, aiming to challenge interpretations and reduce researcher bias.

An audit trail was established by meticulously documenting the research process, including interview transcripts, coding decisions, reflective notes, and steps in thematic analysis, to ensure transparency and facilitate replication.

Member checking was conducted by inviting participants to review and verify the accuracy of their transcripts and preliminary themes, thereby ensuring the credibility of our interpretations.

Any discrepancies in coding or theme development were resolved through discussion among the research team until consensus was achieved. This rigorous approach ensured that the findings accurately represented the complexity and diversity of nurses’ lived experiences.

**Exploring the nature of lived experience**: This stage focused on precisely defining the phenomenon under investigation and elucidating the researcher’s relationship to it. The emphasis was on the lived experiences of home psychiatric nurses, highlighting their challenges, emotional resilience, and professional development. During this phase, the researcher engaged in critical self-reflection to identify and set aside pre-existing assumptions and potential biases, thereby fostering an open and exploratory analytical approach.

**Examining experience as it is lived**: This phase involved collecting detailed, first-person accounts of lived experiences without imposing predefined categories. In-depth semi-structured interviews were conducted with home psychiatric nurses, allowing participants to freely articulate their experiences in their own words. Nurses recounted real-life situations, emotional reactions, and coping strategies related to their professional roles. Field notes and observational insights were also incorporated to enhance the contextual understanding of nurses’ daily work realities.

**Reflecting on the Core Themes**: In this phase, essential themes were identified and interpreted using Interpretative Phenomenological Analysis (IPA). The data were examined iteratively to reveal recurring patterns and meanings related to occupational challenges, resilience-building strategies, and professional growth. Core themes—such as emotional burden, nurse–patient relationships, coping mechanisms, and professional development—were systematically extracted and analytically explored.

**Writing and Rewriting**: This phase underscored the role of writing as an interpretive act central to phenomenological inquiry. Findings were presented through rich narrative accounts supported by verbatim participant quotations to preserve participants’ voices. Interpretive writing was employed to situate individual experiences within broader nursing and theoretical frameworks, including Resilience Theory and Transition Theory. Reflexive writing practices were maintained throughout to minimize researcher bias and ensure fidelity to participants’ lived meanings.

**Maintaining a Strong and Oriented Relation to the Phenomenon**: The analysis remained closely aligned with the essence of home psychiatric nursing, avoiding unnecessary abstraction or detachment from practice. Interpretations were grounded in the real-world context of home-based mental health care, ensuring that findings were meaningful, practical, and relevant. A clear and consistent connection to the study objectives was maintained to prevent superficial or overly theoretical interpretations.

**Balancing the Research Context by Considering Parts and the Whole**: This phase ensured a holistic understanding of the phenomenon by examining data at both individual and collective levels. Personal narratives were analyzed alongside cross-case thematic patterns to generate an integrated and coherent interpretation. Relationships among themes were critically examined to ensure logical consistency and conceptual clarity. Credibility was further enhanced through peer debriefing and cross-checking interpretations. NVivo software was utilized to support the organization, coding, and systematic analysis of qualitative data, facilitating the identification of salient themes related to challenges, emotional resilience, and professional development in home psychiatric nursing.

## Results

Table 1 presents data from a study involving 20 psychiatric nurses employed in home health care (HHC). The participants’ ages ranged from 22 to 55 years, with an average age of 34.2 ± 8.5 years, suggesting a workforce that is relatively young to mid-career. The majority were female (60%) and Saudi nationals (75%), mirroring the local nursing workforce demographics. In terms of educational background, most held a bachelor’s degree (70%), while a smaller portion had a diploma (15%) or postgraduate qualifications (10%), consistent with the professional standards for psychiatric nursing in Saudi Arabia. A significant number of participants were married (60%), and most resided in urban areas (70%). Regarding professional experience, the nurses had an average of 8.6 ± 5.2 years in psychiatric nursing, with 6.9 ± 4.1 years specifically in HHC, indicating moderate experience in both general psychiatric and home-based care. About 60% had chosen to work in HHC voluntarily, while 35% were in obligatory roles. The nurses primarily visited urban patient areas (65%) and conducted an average of 6.3 ± 2.8 visits per week, indicating a manageable patient load. The psychiatric disorders encountered were varied, with mood disorders being the most common (30%), followed by psychotic (25%) and anxiety disorders (20%). Lastly, 60% of the nurses had received prior training before working in HHC, suggesting that nearly half might require additional orientation or professional development to improve patient care. Overall, the sample represents a moderately experienced, predominantly female, urban-based HHC nursing workforce with diverse exposure to psychiatric disorders. The inclusion of the “Not stated” category ensures transparency regarding missing data, which is crucial for accurate interpretation.

**Table 1:** Demographic characteristics of the participants (*N* = 20).

Variable	Category	n (%)	Mean ± SD
Age (years)	20–29	5 (25)	34.2 ± 8.5
	30–39	8 (40)	—
	40–49	4 (20)	—
	≥50	2 (10)	—
	Not stated	1 (5)	—
Gender	Male	7 (35)	—
	Female	12 (60)	—
	Not stated	1 (5)	—
Nationality	Saudi	15 (75)	—
	Non-Saudi	4 (20)	—
	Not stated	1 (5)	—
Educational level	Diploma	3 (15)	—
	Bachelor	14 (70)	—
	Master/PhD	2 (10)	—
	Not stated	1 (5)	—
Marital status	Single	5 (25)	—
	Married	12 (60)	—
	Divorced/Widowed	2 (10)	—
	Not stated	1 (5)	—
Residence area (participants)	Urban	14 (70)	—
	Rural	5 (25)	—
	Not stated	1 (5)	—
Years of experience in psychiatric nursing	<5	4 (20)	8.6 ± 5.2
	5–10	8 (40)	—
	>10	7 (35)	—
	Not stated	1 (5)	—
Years of experience in home health care (HHC)	<5	6 (30)	6.9 ± 4.1
	5–10	10 (50)	—
	>10	3 (15)	—
	Not stated	1 (5)	—
Work type	Obligatory	7 (35)	—
	Freely selected	12 (60)	—
	Not stated	1 (5)	—
Residence area of patients visited	Urban	13 (65)	—
	Rural	6 (30)	—
	Not stated	1 (5)	—
Number of visits per week	1–5	8 (40)	6.3 ± 2.8
	6–10	9 (45)	—
	>10	2 (10)	—
	Not stated	1 (5)	—
Types of psychiatric disorders	Mood disorders	6 (30)	—
	Psychotic disorders	5 (25)	—
	Anxiety disorders	4 (20)	—
	Other	3 (15)	—
	Not stated	2 (10)	—
Previous training before HHC	Yes	12 (60)	—
	No	6 (30)	—
	Not stated	2 (10)	—

**Figure 3:**
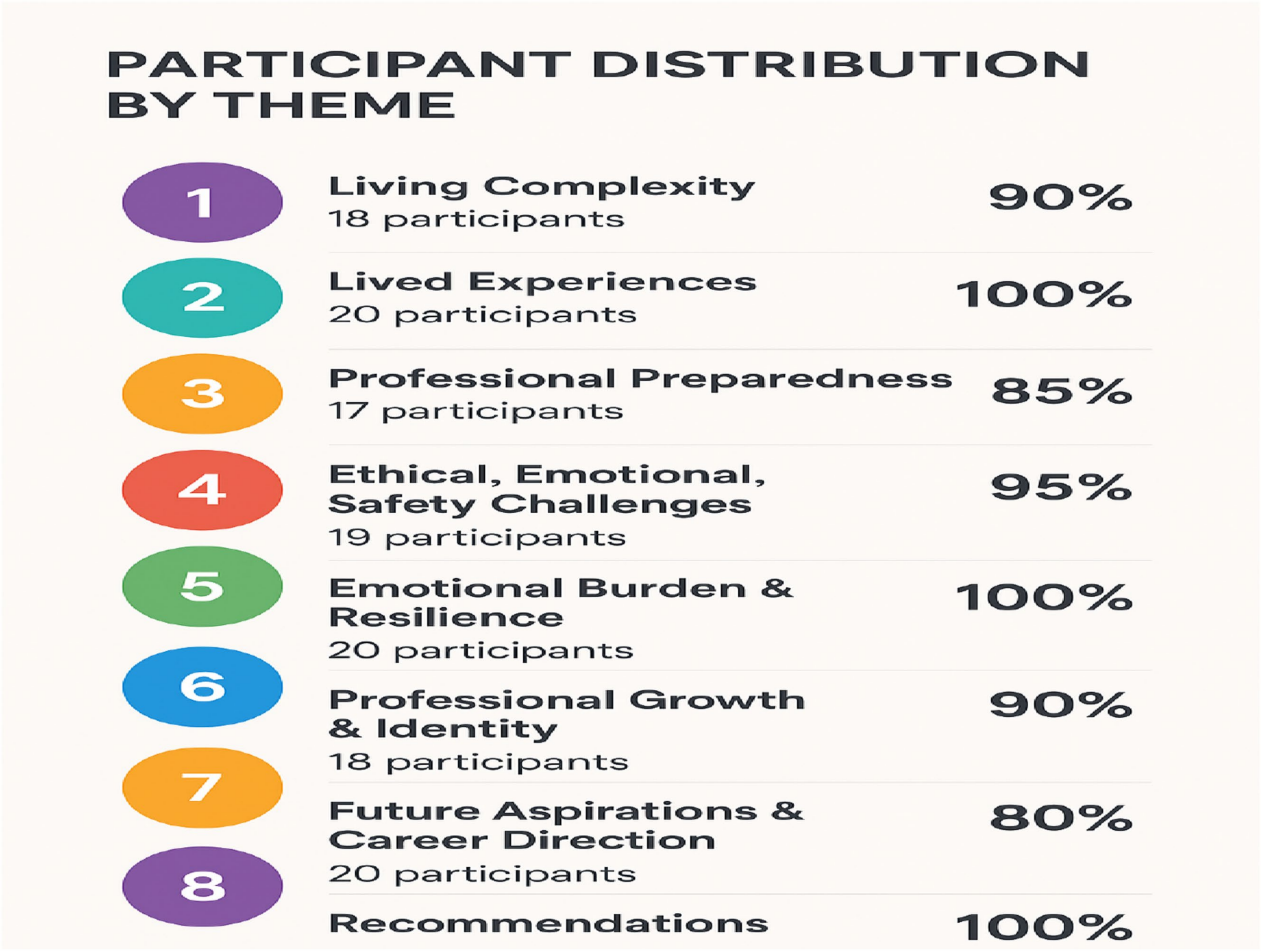
Number of participants contributing to each theme. N = 20.

Figure 3 presents a summary of how frequently and what percentage of participants contributed to each theme in this qualitative study, which included 20 participants. The data shows strong representation across all themes, highlighting their significant relevance to the participants’ experiences. These frequencies and percentages are used solely for descriptive purposes to enhance transparency, and in support of the interpretive narrative in accordance with qualitative research methods guidance in the literature [e.g., (37)].

Theme 1—Living Complexity (90%) indicates that a large majority (18 out of 20) discussed complex personal, social, and environmental challenges, emphasizing the role of complexity in their lives. Theme 2—Lived Experiences (100%) and Theme 5—Emotional Burden & Resilience (100%) were mentioned by all participants, underscoring the importance of sharing personal experiences and handling emotional challenges in understanding the phenomenon. Theme 3—Professional Preparedness (85%) suggests that most participants felt professionally capable, though some gaps in preparation were noted. Theme 4—Ethical, Emotional, Safety Challenges (95%) points to the frequent occurrence of ethical dilemmas, emotional stress, and safety concerns in participants’ work, aligning with literature on high-stakes professional settings. Theme 6—Professional Growth & Identity (90%) highlights the importance of career development and self-concept, while Theme 7—Future Aspirations & Career Direction (80%) indicates that although not all participants had clear goals, career aspirations remain significant. Theme 8—Recommendations (100%) reflects the participants’ strong desire to provide suggestions for improvements, emphasizing the study’s practical and action-oriented nature. Overall, themes with full representation are central to participants’ experiences, while those with slightly lower representation suggest variability, offering opportunities to explore differences. The overall high percentages indicate robust data saturation, supporting the validity of the thematic framework.

### Qualitative findings and main codes of lived experiences of home-based psychiatric nursing

**Figure 4:**
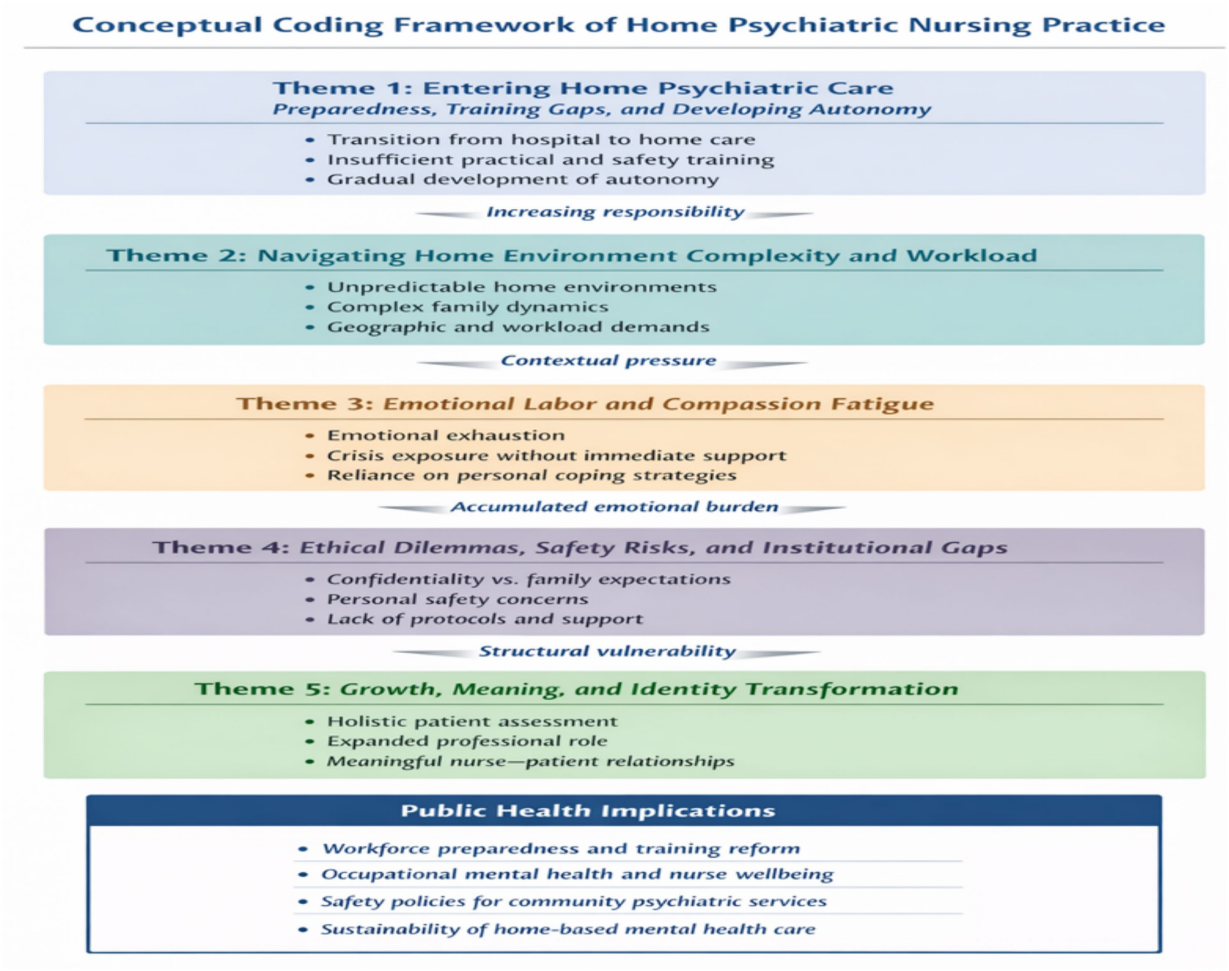
Coding framework of lived home experiences.

Figure 4 visually synthesizes the qualitative coding framework, developed in line with qualitative research principles by Braun and Clarke,and Van Manen’s phenomenological method, which encompasses a range of analytic codes. Each code is defined by a specific operational meaning, criteria for application, conditions for exclusion, participant quotes for illustration, and its connection to a particular subtheme and the related main theme. Each code identifies a significant unit within the data. For example, “Unpredictable routine” highlights the changing nature of home-based psychiatric nursing work and is used when participants discuss variability or uncertainty in daily tasks, but not when referring to consistent hospital routines. Similarly, “Environmental influence” captures instances where the home environment impacts care delivery and is applied when participants mention family dynamics or living conditions; it is not used when discussing factors solely related to the patient. Codes like “Physical risk” or “Confidentiality conflict” address ethical and safety issues, applied when nurses describe aggression, fear, or moral tension, but not when discussing general workload stress. Emotional aspects are captured through codes such as “Emotional fatigue,” used when participants describe emotional heaviness, and “Personal coping,” which reflects strategies like journaling or prayer. Professional growth is represented by codes like “Expanded assessment skills” or “Independence, applied when participants reflect on skill development or a strengthened identity. Future-oriented insights include codes such as “Sense of meaning,” applied when participants express a commitment to home care, and “Fatigue-driven reconsideration,” used when describing a desire to return to hospital settings due to exhaustion. Finally, system- or organizational-level improvements are captured through codes like “Need for structured training” and “Support systems,” which reflect participants’ recommendations for professional development and institutional support. Each code is thus directly connected to the subtheme and main theme it supports, contributing to a structured, rigorous, and transparent analytic framework. Collectively, the figure highlights the dynamic interplay between individual, organizational, and system-level factors, underscoring critical public health implications for workforce preparedness, occupational mental health, and the sustainability of home-based psychiatric services (Table 2).

**Table 2:** Summary of themes and illustrative quotes on home-based psychiatric nursing.

Theme	Analytical focus	Illustrative quotes
Theme 1: Entering home psychiatric care—preparedness, training gaps, and developing autonomy	Transition from hospital-based psychiatry to home care revealed insufficient preparation for safety assessment, crisis management, and environmental evaluation. Over time, nurses developed autonomy, confidence, and independent clinical judgment.	“I have worked in the hospital for 7 years, but visiting patients at home is completely different.” (P3) “The training we received was short and mostly theoretical.” (P6) “Home care forces you to be more independent. You make decisions on the spot.” (N2)
Theme 2: Navigating the complexity of the home environment and workload demands	Home visits exposed nurses to unpredictable environments, complex family dynamics, and diverse psychiatric conditions, compounded by high workload intensity and geographic challenges.	“At home, you see the whole story—the stress, the family tension.” (P7) “Every house is different… sometimes it makes everything harder.” (N4) “Rural visits take much longer, which is exhausting.” (P2)
Theme 3: Emotional labor, compassion fatigue, and carrying the weight of care	Sustained exposure to patient suffering and crises without immediate support resulted in emotional exhaustion and compassion fatigue, prompting reliance on personal coping strategies.	“I left one home and sat in my car crying.” (P6) “During withdrawal, the aggression can escalate quickly, and you have no backup.” (N3) “After hard visits, I write everything in my journal.” (P18)
Theme 4: Ethical dilemmas, safety risks, and the absence of institutional support	Nurses faced ethical conflicts related to confidentiality and family expectations, alongside safety risks in isolated settings and a lack of organizational backing or clear protocols.	“How do I respect the patient and still keep peace with the family?” (P8) “A patient suddenly became aggressive… I thought, ‘What if I cannot get out?’” (P11) “At home, you do not have a team. No doctor nearby.” (P1)
Theme 5: Growth, meaning, and identity transformation in home psychiatric nursing	Despite challenges, nurses experienced professional growth, expanded roles, and deep personal fulfillment through meaningful patient relationships and holistic care.	“I learned to assess not just the patient, but the whole ecosystem.” (P7) “In home care, you become more than a nurse.” (P5) “When a patient tells you, ‘Your visit is the best part of my week,’ you know you cannot leave.” (P9)

### Themes of the study

Analysis of the interview data revealed five interrelated themes that collectively describe nurses’ lived experiences of providing psychiatric care in home-based settings. These themes reflect a dynamic interplay between professional preparedness, environmental complexity, emotional labor, ethical and safety challenges, and personal professional growth.

#### Theme 1: Entering home psychiatric care—preparedness, training gaps, and developing autonomy

Participants consistently described entering home psychiatric nursing with moderate hospital-based psychiatric experience yet emphasized that this background did not fully prepare them for the realities of home-based care. While many felt clinically competent in symptom management, the unpredictability and isolation of home visits demanded skills that extended beyond traditional hospital training.

“I have worked in the hospital for 7 years, but visiting patients at home is completely different. You face unexpected situations that we never see in the ward” (P3).

Nurses reported significant gaps in formal preparation, particularly in safety assessment, crisis management, and evaluating home environments. Orientation programs were perceived as brief and overly theoretical, leaving nurses to rely on experiential learning.

“The training we received was short and mostly theoretical. I wish we had more practical guidance for home care” (P6).

“We were never taught how to assess risk in someone’s home. You learn as you go” (N5).

Despite these gaps, participants described a gradual development of autonomy and confidence over time. Working independently in unpredictable contexts fostered rapid decision-making and strengthened clinical judgment.

“Home care forces you to be more independent. You make decisions on the spot” (N2).

This transition from initial uncertainty to professional self-reliance marked an important stage in nurses’ adaptation to home psychiatric care. These early experiences of uncertainty and reliance on experiential learning laid the foundation for later divergence in nurses’ confidence and role identity, as described in Theme 5.

#### Theme 2: Navigating the complexity of the home environment and workload demands

Home visits exposed nurses to layers of psychosocial and environmental complexity that were largely invisible in hospital settings. Participants described entering patients’ personal worlds, where family dynamics, environmental hazards, and limited resources shaped care delivery.

“At home, you see the whole story—the stress, the family tension, the real issues behind the patient’s behavior” (P7).

“Every house is different… sometimes the environment supports care, other times it makes everything harder” (N4).

In addition to environmental variability, nurses managed a diverse patient population with wide-ranging psychiatric diagnoses, requiring constant adaptation of clinical approaches.

“One day I might see a patient with severe depression, and the next day someone with schizophrenia—each visit requires a different approach” (P7).

Workload intensity further compounded these challenges. Geographic dispersion, back-to-back visits, and time constraints limited opportunities for documentation, reflection, and recovery.

“Visiting 8–10 patients a week is doable in urban areas, but rural visits take much longer, which is exhausting” (P2).

“Sometimes there’s barely time to document notes properly” (P14).

These findings highlight how environmental, and workload demands collectively intensified the complexity of home psychiatric nursing.

#### Theme 3: Emotional labor, compassion fatigue, and carrying the weight of care

Participants described the emotional burden of home psychiatric care as profound and enduring. Nurses often encountered patient crises, aggression, and deep suffering without immediate professional backup, amplifying stress and emotional vulnerability.

“During withdrawal, the aggression can escalate quickly, and you have no backup, unlike in the hospital” (N3).

“I left one home and sat in my car crying. The patient’s sadness felt too heavy. I could not leave it behind” (P6).

Unlike hospital settings, emotional exposure did not end when the visit concluded; many nurses described carrying patients’ distress with them long after leaving the home. This ongoing emotional engagement contributed to compassion fatigue and emotional exhaustion.

Despite this burden, nurses actively developed personal coping strategies, including journaling, mindfulness, and reflection, to sustain themselves emotionally.

“After hard visits, I write everything in my journal. It helps me release the weight” (P18).

“You learn to breathe deeply, to remind yourself that you are doing your best” (P10).

However, participants emphasized that personal resilience alone was insufficient without institutional emotional support.

#### Theme 4: Ethical dilemmas, safety risks, and the absence of institutional support

Ethical challenges were a recurring feature of home-based psychiatric care, particularly when nurses navigated conflicts between patient autonomy and family expectations.

“The family wanted me to hide details from the patient. I stood there thinking, ‘How do I respect the patient and still keep peace with the family?’” (P8).

Safety concerns were equally prominent. Nurses reported feelings of vulnerability when encountering aggression or volatile situations in isolated settings.

“A patient suddenly became aggressive. I was alone in the room. For a moment, I thought, ‘What if I cannot get out?’” (P11).

Participants consistently contrasted home care with hospital environments, emphasizing the absence of immediate backup, security, or interdisciplinary support.

“At home, you do not have a team. No doctor nearby. No security. No quick backup. It’s just you” (P1).

This lack of organizational infrastructure heightened perceptions of risk and reinforced calls for clear safety protocols, supervision, and institutional backing.

#### Theme 5: Growth, meaning, and identity transformation in home psychiatric nursing

Despite the substantial clinical, emotional, and ethical challenges described in earlier themes, participants consistently framed home psychiatric nursing as a powerful site of professional growth and identity transformation. Rather than being defined solely by adversity, nurses described how sustained exposure to autonomous practice reshaped their understanding of their professional roles.

Participants highlighted that the holistic nature of home-based care expanded their responsibilities beyond symptom management to include assessment of family dynamics, environmental risks, and psychosocial resources:

“I learned to assess not just the patient, but the whole ecosystem—family, environment, everything.” (P7).

For many nurses, working independently fostered adaptability, leadership, and clinical confidence:

*“In home care, you become more than a nurse. You’re a guide, a problem-solver, a listener.”* (P5).

One nurse voiced uncertainty about role expectations, saying, “*At times, I’m unsure if my role is solely to administer medication or also to provide emotional support to the family. The role is not always clear*.” Conversely, another participant demonstrated a more established sense of professional identity, stating, *“Over time, I’ve learned to balance safety, care, and professionalism. Now, I feel confident in my role during home visits.”*

While participants encountered similar challenges in home psychiatric nursing, there were significant differences in how nurses perceived and managed these situations. Nurses with more extensive experience often reported feeling more assured and having well-defined role boundaries, whereas those with less experience expressed confusion about their responsibilities and professional identity. Participants conveyed varying degrees of clarity about their professional roles. However, identity transformation was not experienced uniformly. Nurses with longer professional experience described a more consolidated and confident role identity, marked by clear boundaries and decisiveness in complex situations:

*“Over time, I’ve learned how to balance safety, care, and professionalism. Now, I feel confident in my role during home visits.”* (P11).

In contrast, less experienced nurses reported ongoing role ambiguity, particularly in navigating expectations related to emotional support, family involvement, and clinical responsibility:

*“Sometimes I’m not sure if my role is only medication or also supporting the family emotionally.”* (P3).

While the work was frequently described as meaningful and purpose-driven, the emotional demands of sustained engagement led some participants to question long-term career sustainability:

*“I love the work, but the emotional exhaustion is real. Some days I think of going back to the hospital.”* (P2).

Overall, this theme illustrates how home psychiatric nursing functions as both a site of professional fulfillment and emotional strain, with role identity formation evolving differently depending on nurses’ experience and capacity to integrate autonomy, responsibility, and self-care.

### Analytical summary

This study reveals the dual reality of home-based psychiatric nursing: a practice characterized by complexity, emotional intensity, ethical tension, and safety risks, yet simultaneously marked by professional autonomy, personal growth, and meaningful patient relationships. Nurses’ narratives underscore the urgent need for specialized training, structured organizational support, safety protocols, workload management, and emotional support systems to sustain nurses in this demanding but profoundly impactful field.

## Discussion

### Navigating the complexity of the home environment and workload demands

This study explored the lived experiences of home psychiatric nurses in Jeddah, Saudi Arabia, with a focus on professional challenges, emotional resilience, and professional identity development within home-based mental healthcare. The demographic profile of the participants reflects a moderately experienced psychiatric nursing workforce that is predominantly female and largely educated at the bachelor’s level, which is consistent with national workforce trends in Saudi mental health nursing (38, 39).

Participants’ years of experience in both psychiatric and home health nursing situate them at a professional stage characterized by increasing autonomy alongside heightened responsibility. Unlike inpatient psychiatric settings, home-based care requires nurses to independently manage environmental risks, patient behavior, and family interactions, reinforcing findings from international literature that community psychiatric nursing demands distinct competencies related to autonomy, safety assessment, and emotional regulation (40). These contextual characteristics are essential for understanding the emotional and professional challenges identified in this study.

### Entering home psychiatric care preparedness, training gaps, and developing autonomy

The findings indicate that transitioning from hospital-based psychiatric care to home settings exposes notable gaps in professional preparedness. Despite prior psychiatric experience, nurses reported challenges related to crisis management, safety evaluation, and managing severe mental disorders, echoing evidence that community psychiatric roles often exceed the scope of traditional clinical training (6, 41, 42).

Family involvement emerged as a complex and influential factor, functioning both as a facilitator of care and a source of emotional strain. This dual role of families has been documented in previous studies, which emphasize that unrealistic expectations and insufficient mental health literacy can increase nurses’ emotional burden (43, 44). Compared with inpatient settings—where care is more structured and institutionally supported—home psychiatric care places greater responsibility on nurses to negotiate family dynamics independently (45).

### Emotional labor, compassion fatigue, and carrying the weight of care

The emotional demands identified in this study align with the Job Demands–Resources (JD-R) theory, which posits that occupational well-being depends on the balance between job demands and available resources (46). High job demands in home psychiatric nursing—including unpredictable environments, emotional labor, and safety concerns—combined with limited organizational resources contributed to emotional exhaustion, consistent with findings among psychiatric nurses in Saudi Arabia and similar contexts (47, 48).

Role Strain Theory further explains how overlapping responsibilities and unclear professional boundaries intensify stress when institutional guidance is limited (49). However, nurses in this study also developed adaptive personal and relational resources—such as autonomy, emotional regulation, and therapeutic confidence—that supported resilience. This dynamic interaction between occupational strain and resource development mirrors international findings on resilience among community mental health nurses (16, 49).

### Professional identity formation and divergent trajectories in home psychiatric nursing

The results of this research emphasize that forming a professional identity is a key and evolving process in home psychiatric nursing, influenced by factors such as autonomy, emotional challenges, and the organizational environment of the workplace. Although nurses face common occupational challenges in home-based psychiatric care, the study revealed both similarities and differences in how they negotiate and maintain their professional identity. This research expands on the existing literature by not only focusing on burnout prevalence but also shedding light on the processes through which identity is either consolidated or fragmented in community mental health practice (50, 51). Consistent with previous studies, nurses who experienced higher levels of autonomy and role recognition reported greater professional confidence and a more solidified sense of professional identity (39, 52).

In this study, ongoing exposure to independent decision-making and comprehensive care seemed to promote professional growth and meaning making, reinforcing nurses’ dedication to home psychiatric practice. These findings support the theoretical perspectives that view professional identity as an evolving construct shaped through practical experience rather than formal training (53). However, this study also highlighted significant differences in professional paths. Some nurses, especially those with less experience or limited organizational support, reported ongoing role ambiguity, emotional fatigue, and uncertainty regarding long-term career sustainability. This divergence aligns with regional and international evidence suggesting that unclear role boundaries and inadequate institutional support contribute to emotional exhaustion and professional disengagement among psychiatric and community mental health nurses (54, 55). The interaction between patient behavior, family involvement, and organizational infrastructure further influenced the development of nurses’ professional identity. Nurses who perceived sufficient supervisory support and clear safety frameworks were more likely to report resilience, confidence and professional fulfillment. Conversely, those who felt unsupported in managing complex family dynamics or aggressive behaviors were more susceptible to emotional strain and identity fragmentation. This supports previous findings that emphasize the critical role of organizational support in reducing burnout and fostering professional identity consolidation in community mental health settings (56).

Overall, this study illustrates that home psychiatric nursing serves as both a site of professional transformation and as a potential vulnerability. The identified divergent identity trajectories highlight the need for organizational strategies that promote role clarity, mentorship, emotional support, and ongoing professional development to enhance nurses’ resilience and long-term engagement in home-based psychiatric care.

### Ethical dilemmas, safety risks, and the absence of institutional support

Organizational support emerged as a decisive factor influencing nurses’ emotional resilience, professional identity, and workforce retention. Participants emphasized the importance of structured supervision, clear safety protocols, ongoing training in crisis management, and effective communication pathways with specialist services. These findings are consistent with recommendations from the World Health Organization (57) and supported by evidence indicating that systemic support reduces role strain and emotional labor in community mental health settings (58, 59).

By shifting attention from individual coping strategies to organizational and system-level conditions, this study aligns with global calls to strengthen community mental health infrastructure and support the sustainability of home-based psychiatric nursing services (60).

### Novel contribution of the study

This study adds novel insights by illuminating how emotional resilience and professional identity are actively constructed within the context of home psychiatric nursing in Saudi Arabia—an underexplored area in regional and international literature. Unlike prior research that emphasizes workload or burnout, this study reveals divergent identity trajectories shaped by autonomy, family dynamics, and organizational support. By foregrounding nurses’ lived experiences in home-based psychiatric care, the findings contribute to a more nuanced understanding of workforce sustainability, resilience, and professional growth in community mental health nursing.

### Public health implications

Strengthening formal job resources—including structured training, supervision, safety protocols, and clear role definitions—can mitigate role strain and enhance resilience. From a public health perspective, supporting home psychiatric nurses is critical for workforce sustainability, patient safety, and the quality of community-based mental health services.

### Limitations

This research has various limitations that must be considered when analyzing the results. Initially, it was carried out in the only psychiatric hospital in Jeddah, which might restrict applicability to other areas or healthcare environments. Secondly, while data saturation was reached, the number of nurses involved was limited compared to the extensive patient population, potentially limiting the variety of viewpoints collected. Third, in this context, care at home mainly involved administering injections rather than offering complete, multidisciplinary services, so the participants’ experiences might not entirely capture the challenges and opportunities of optimal home psychiatric care.

Moreover, employing a phenomenological perspective centered on nurses’ personal experiences instead of institutional frameworks or regulations. Although this offers useful understandings of personal viewpoints, it fails to consider how organizational elements influence service provision. Future studies might investigate these organizational aspects to enhance the existing findings and provide a more thorough insight into home psychiatric nursing.

## Conclusion

Home psychiatric care in Jeddah, Saudi Arabia, presents both deep opportunities and major challenges. Nurses experience professional growth, increased autonomy, and identity change while navigating a complex psychosocial environment, ethical dilemmas, family dynamics, and security issues. Emotional resilience and adaptive coping strategies allow them to cope with these challenges, but organizational support and specialized training are essential to maintain the efficiency and welfare of the workforce. These findings highlight the need for systemic interventions at the educational, organizational, and policy levels to optimize home psych care and support the nurses providing it.

### Implications

**Practice**: Home psych care requires skills beyond hospital level, including advanced psychosocial assessment, risk management, and family-oriented communication.

**Education**: Nursing curricula should incorporate community mental health training, crisis intervention, and experiential learning in home settings to strengthen preparedness.

**Policy and Management**: Organizations must establish clear role definitions, safety protocols, supervision structures, and structured referral pathways to support nurses’ autonomy and reduce emotional labor.

**Research**: Future studies should explore interventions that enhance resilience, professional identity, and family engagement strategies in home-based psychiatric care.

### Recommendations

- Develop and implement specialized training programs focusing on home psychiatric care, including crisis management, safety assessment, and complex psychiatric disorders.
- Provide structured supervision and debriefing sessions to support emotional resilience and professional reflection.
- Create clear communication and referral pathways between community nurses and mental health specialists to enhance care continuity.
- Offer family psychoeducation programs to align expectations, reduce conflict, and strengthen collaborative care.
- Encourage reflective practice and mindfulness interventions to cultivate emotional resilience and mitigate role strain.
- Establish protocols to ensure nurse safety during home visits and provide emotional support to manage stress and prevent burnout.
- Advocate for policies that recognize the complexity of home psychiatric nursing and allocate sufficient resources to enable sustainable, high-quality care.
- The findings also indicate several directions for future research: Longitudinal studies tracking changes in resilience, professional identity, and preparedness over time, and comparative studies across different institutions or regions to examine contextual influences.

## Data Availability

The raw data supporting the conclusions of this article will be made available by the authors, without undue reservation.
