# Supplementary material for: Understanding home psychiatric nursing: a phenomenological study on nurses’ challenges, emotional resilience, and professional growth in Jeddah, Saudi Arabia
Source: Front Public Health. 2026 Mar 9;14:1775451. doi: 10.3389/fpubh.2026.1775451 (PMC13007937; doi:10.3389/fpubh.2026.1775451)
Supplement: Supplementary file 1 [file Supplementary_file_1.pdf]

Tool of the study:

Final Interview Guide

Section 1: Demographic Data

1. **Age**: ____ years
2. **Gender**: ☐ Male ☐ Female ☐ Other (specify)
3. **Nationality**: __________
4. **Educational Qualification**:

   ☐ Diploma ☐ Bachelor’s ☐ Master’s ☐ Doctorate ☐ Other (specify)
5. **Marital status:** single ☐ Married☐ divorced ☐widowed ☐
6. **Residence area: Urban ☐ Rural ☐**
7. **Years of Experience in Nursing**: ____ years
8. **Years of Experience in Home Psychiatric Nursing**: ____ years
9. **Current Workplace/Organization (optional)**: __________
10. **Current patients’ residence area** : urban ☐ rural☐
11. **Number of Patients Managed Per Week**: ____
12. **Types of Psychiatric Conditions Commonly Managed** (e.g., schizophrenia, bipolar disorder, depression, substance use disorder, etc.): __________
13. **Have you received any specialized training in home psychiatric care?** ☐ Yes ☐ No (If yes, specify) __________

Section 2: In-Depth Interview Questions

1. Exploring the Lived Experience (Phase 1 & 2: Turning to the Nature of the Lived Experience & Investigating Experience as We Live It)

1. Can you describe a **typical day** as a home psychiatric nurse?
2. What led you to choose **home-based psychiatric nursing** over hospital-based care?
3. Can you recall a **memorable patient experience** that shaped your perspective on your role?

2. Challenges in Home Psychiatric Nursing (Phase 3: Reflecting on the Essential Themes)

4. What are the **biggest challenges** you face in delivering psychiatric care in home settings?
5. How do you **navigate ethical dilemmas** when providing care at a patient’s home?
6. Have you ever felt **physically or emotionally unsafe** in your work? Can you share an experience?
7. What are the **biggest barriers** to effective care in a home setting compared to hospital settings?

3. Emotional Resilience and Coping Mechanisms (Phase 3 & 4: Reflecting on Themes & Writing and Rewriting)

8. How do you manage the **emotional burden** of caring for psychiatric patients in a home environment?
9. What strategies help you **stay resilient** despite the stress of your job?
10. Have you developed **personal coping mechanisms** to deal with challenging cases?
11. Can you recall a situation where you felt emotionally **overwhelmed** and how you handled it?
12. Do you receive **institutional or peer support** for emotional challenges at work? If not, what kind of support would help?

4. Professional Growth and Role Adaptation (Phase 5: Maintaining a Strong and Oriented Relation to the Phenomenon)

13. How has working in home psychiatric nursing **shaped your professional identity**?
14. What skills have you **gained or strengthened** through this experience?
15. Have you noticed any **differences in your approach** to psychiatric nursing since transitioning to home care?
16. What do you think makes a nurse **well-suited** for home psychiatric nursing?

5. Role Transition and Career Aspirations (Phase 5 & 6: Maintaining a Strong Relation & Considering Parts and the Whole)

17. How has your **understanding of psychiatric care evolved** through your experiences?
18. If given the chance, would you **continue working in home psychiatric care or transition to another setting**? Why?
19. How do you think your **career has been shaped** by this role?

6. Recommendations and Final Reflections (Phase 6: Balancing the Whole and the Parts)

20. What improvements do you believe are needed to **support home psychiatric nurses**?
21. If you could tell **new nurses entering this field one key lesson**, what would it be?
22. Is there anything **we haven’t discussed** that you think is important about your experience?

"Thank you for sharing your experiences and insights. Your contributions are invaluable in understanding the challenges and opportunities in home psychiatric nursing. Before we conclude, is there any final thought or reflection you’d like to share?"
